# Women with Endometriosis Are More Likely to Suffer from Migraines: A Population-Based Study

**DOI:** 10.1371/journal.pone.0033941

**Published:** 2012-03-19

**Authors:** Meng-Han Yang, Peng-Hui Wang, Shuu-Jiun Wang, Wei-Zen Sun, Yen-Jen Oyang, Jong-Ling Fuh

**Affiliations:** 1 Department of Computer Science and Information Engineering, National Taiwan University, Taipei City, Taiwan, Republic of China; 2 Department of Obstetrics and Gynecology, Taipei-Veterans General Hospital, Taipei City, Taiwan, Republic of China; 3 Faculty of Medicine, National Yang-Ming University School of Medicine, Taipei City, Taiwan, Republic of China; 4 Department of Neurology, Neurological Institute, Taipei-Veterans General Hospital, Taipei City, Taiwan, Republic of China; 5 Brain Research Center, National Yang-Ming University Schools of Medicine, Taipei City, Taiwan, Republic of China; 6 Department of Anesthesiology, National Taiwan University Hospital, Taipei City, Taiwan, Republic of China; 7 Graduate Institutes of Biomedical Electronics and Bioinformatics, National Taiwan University, Taipei City, Taiwan, Republic of China; Brigham & Women's Hospital, and Harvard Medical School, United States of America

## Abstract

Previous research suggests that a co-morbid relationship exists between migraine and endometriosis; however, results have been inconsistent. In addition, female hormones, which are important in the pathogenesis and management of endometriosis, have been reported to precipitate migraine attacks and may confound the results. The aim of this population-based cohort study was to explore the relationship between migraine and endometriosis in women of reproductive age (18–51 years). Data were derived from the National Health Insurance Research Database of Taiwan, which contains outpatient and inpatient records from 2000 to 2007. Our study cohort included 20,220 endometriosis patients and 263,767 controls without endometriosis. We analyzed the prevalence of migraine in these women as recorded during the eight years of the database. Our results found that patients with endometriosis were more likely to suffer migraine headaches compared to controls (odds ratio [OR], 1.70; 95% confidence interval [CI] [1.59, 1.82]; *p*<0.001). In addition, the co-morbid association between migraine and endometriosis remained significant after the data were controlled for age and frequently utilized hormone therapies (OR, 1.37; 95% CI, [1.27, 1.47]; *p*<0.001). The results of this cohort study support the existence of a co-morbid relationship between migraine and endometriosis, even after adjusting for the possible effects of female hormone therapies on migraine attacks.

## Introduction

Endometriosis is a gynecological disorder defined by the presence of viable, extrauterine, endometrial tissue. This tissue arises only in women of menstrual age, can grow or bleed cyclically, and may cause adhesions. Typical symptoms include pelvic pain, dysmenorrhea, and infertility [1]. Endometriosis affects 6–10% of women of reproductive age, 50–60% of women and teenage girls with pelvic pain, and up to 50% of women with infertility [2].

Migraine is a neurological disorder that also commonly occurs in women of reproductive age. An important advance in the standardization of migraine diagnosis was the publication of operational criteria by the Headache Classification Committee of the International Headache Society (IHS) in 1988 [3]. Physicians diagnose migraine according to the clinical symptoms. Migraines typically present as recurrent episodic headaches and frequently have the following pain characteristics: a unilateral location, a pulsating quality, moderate-to-severe intensity, and aggravation by routine physical activity. In addition, the attacks may be associated with nausea, photophobia, or phonophobia symptoms.

Endometriosis and migraine share many similarities in epidemiology, pathogenesis, and the physical or psychiatric co-morbidities that can accompany them [4], [5], [6], [7], [8]. For example, early menarche, a well-known risk factor for endometriosis, is also associated with an increased risk of migraine [9]. Similarly, menorrhagia, a frequent complaint of women with endometriosis, is also common in women with migraine, with 63% of migraine patients reporting a recent history of menorrhagia, compared to 37% of controls [10]. In addition, migraine patients with endometriosis are more likely to have mood and pain-related disorders than migraine patients without endometriosis [11].

Men also suffer from migraines; however, both the age-adjusted prevalence of migraine (among men 8% and among women 26%) and age of onset for migraine (among men 20% in the first decennium, and 23% in the second and third decennium; among women 9% in the first decennium, 34% in the second, and 30% in the third) differ significantly between men and women [12], [13]. Female hormones may help explain the sex differences in migraine experience and the incidence of headache disorders.

Cycling female hormones are abundant in women of reproductive age and are deficient in postmenopausal women. As mentioned earlier, these hormones play a key role in the development of endometriosis. Brandes provided evidence linking estrogen to migraines [14], while Misakian et al. reported that hormone therapy for postmenopausal women was associated with an increased incidence of migraines [15]. Conventional oral contraceptives can also exacerbate migraines [16], [17], while drug-induced menopause, which decreases female hormones, can reduce migraine symptoms. Danazol, a well-known male hormone analogue used frequently in the management of endometriosis, has been successful in controlling cyclic migraine symptoms [18]. In a similar vein, progestogen-only oral contraceptives can reduce the frequency of migraine attacks [19]. Taken together, these data suggest that endometriosis and migraines may have a co-morbid relationship due to the association with female hormones.

Although several studies have supported a relationship between endometriosis and migraines, conclusions regarding the association remain unclear. For instance, Tietjen et al. reported that both the frequency and the prevalence of endometriosis were higher in women with migraines than in migraine-free controls [10], [11], and the prevalence of migraines has also been shown to be higher in women with endometriosis than in controls [20]. Nyholt et al. proposed that common genetic influences might underlie the co-morbid relationship between the two diseases [21]. Despite these suggestive data, a recent study by Karp et al. failed to find evidence that migraine headaches were more common in women with endometriosis than in women without the disease [22]. One explanation for these disparate results may be the differences in the size and scope of the study populations used in the various analyses.

The goal of the present study was to explore the relationship between endometriosis and migraines in women of reproductive age. We hypothesized that the migraine prevalence would be higher in women with endometriosis than in women without the disease. To address previous confounding in the literature and to overcome any potential limitations from sample size, we used a large nationwide medical database to investigate the co-morbidity between endometriosis and migraines. Since hormone therapy may be more frequently used in endometriosis patients, we also included information from the database regarding prescriptions for commonly administered hormone regimes, which further isolated the potential confounding effects of these drugs.

## Materials and Methods

The National Health Insurance (NHI) program in Taiwan was initiated in 1995. As of December 2010, it covered over 99% of the population (23,074,487 beneficiaries) and contracted support from almost all medical hospitals and clinics in Taiwan (25,031 institutions) [23]. In 2000, the NHI Bureau initiated a program to construct the NHI Research Database (NHIRD), and to release claims data for academic medical and pathological research. Subsequently, the NHIRD has emerged as one of the largest nationwide medical datasets in the world and has been used extensively for many epidemiological studies. The current study was based on the 2005 NHIRD, which contained the data of 1 million randomly selected patients that were representative of the entire population with regard to age, sex, and insurance cost. Specifically, the database included information regarding both inpatient and outpatient medical claims, as well as prescription records.

### 1. Subjects

The study population was first filtered for ambulatory and inpatient NHIRD claims made between 2000 and 2007 by women of reproductive age (18–51 years old). Disease diagnosis from the database was coded according to the International Classification of Disease, 9^th^ Revision, Clinical Modification (ICD-9 CM).

The case cohort consisted of outpatient, or admitted patients, that had been diagnosed with diseases related to endometriosis by physicians from obstetrics and gynecology departments (ICD-9 CM codes: 617.x). In the end, data from 20,220 endometriosis cases and 263,767 control samples were collected. We also analyzed patients with any ambulatory visits or inpatient record with a diagnosis of migraine, with or without aura (codes: 346.0x, 346.1x, and 346.9x) during the same period.

### 2. Endometriosis symptoms and treatments

For each subject in the study cohort, we searched the database for diagnoses and treatment records related to endometriosis. The diagnosis of endometriosis was mainly based on the clinical symptoms (i.e., dysmenorrhea, which was defined as pelvic pain during, shortly before, or after menstrual periods) medical signs (for example, focal pain or tenderness during pelvic examination), and sometimes imaging (ultrasound) or serum CA125 evaluations [2]. Only those diagnoses that had been made by physicians in an obstetrics and gynecology department were recorded. The diagnostic search criteria included diagnoses related to infertility (ICD-9 CM codes 628.x) and pelvic pain (625.9 and 789.00). Surgical treatments for endometriosis were also recorded, including laparoscopy (ICD-9 CM codes 542.1), laparoscopic lysis of peritoneal adhesions (545.1), laparoscopic oophorotomy (650.1), laparoscopic diagnostic procedures related to ovaries (651.3 and 651.4), laparoscopic local excision or destruction of ovaries (652.3, 652.4, and 652.5), laparoscopic unilateral oophorectomy (653.1), laparoscopic unilateral salpingo-oophorectomy (654.1), laparoscopic removal of the remaining ovary (655.4) and tube (656.4), laparoscopic salpingo-oophoroplasty (657.6), and laparoscopic lysis of adhesions to the ovary and fallopian tube (658.1).

Hormonal treatment commonly used for endometriosis management, including danazol, estrogen, and progestin, was also recorded for each patient. We then analyzed the prescription orders from each drug category for individual subjects.

### 3. Statistical analysis

Analyses were performed using SPSS 18.0 software for Windows (SPSS Inc., Chicago, IL, USA). The chi-square test and Student's T-test were used to compare demographics and clinical variables between endometriosis cases and controls. Logistic regression evaluated the proportion of co-morbid migraines that was related to endometriosis. Two multivariate models were constructed to independently ascertain whether endometriosis had a co-morbid association with migraines. One model assessed this relationship while adjusting for the potential influences of age, infertility, pelvic pain and laparoscopic surgical treatments. The other model was designed to validate the relationship between endometriosis and migraine while adjusting for the potential effects of age and hormone therapies. The results from each model were expressed as an odds ratio (OR) with 95% confidence interval (CI). All tests were two-tailed, and p values<0.05 were considered significant.

## Results

Table 1 summarizes the demographic characteristics of the study population. It should be noted that the endometriosis group was significantly older than the control group. Compared to the controls, the endometriosis patients had a higher incidence of both infertility (14.9 *vs.* 4.8%, respectively; p<0.001) and pelvic pain (43.0 *vs.* 25.7%, respectively; p<0.001). Also as expected, the patients in the endometriosis group were more likely than the controls to have undergone both laparoscopic surgeries (21.2 *vs.* 1.7%, respectively; p<0.001) and danazol treatment (9.2 *vs.* 0.6%, respectively; p<0.001) for the disease. Patients in the endometriosis group were also treated more frequently with female hormones than were the controls, regardless of the type of hormone or its dosage format (respectively: estrogen cream, 1.8 *vs.* 0.4%; estrogen tablet, 35.6 *vs.* 20.7%; progestin injection, 28.7 *vs.* 19.5%; progestin tablet, 48.1 *vs.* 25.9%; all p<0.001).

**Table 1:** Demographic and clinical characteristics of endometriosis patients and controls.

	Endometriosis (*n* = 20,220) (%)	Control (*n* = 263,767) (%)	P-value
Mean age (SD)	38.11 (8.325)	33.94 (9.557)	<0.001
Age (years)			
≤20	318 (1.6)	20,170 (7.6)	<0.001
21–30	4,029 (19.9)	88,358 (33.5)	
31–40	6,745 (33.3)	77,410 (29.3)	
41–50	8,534 (42.2)	71,255 (27.0)	
≥51	594 (2.9)	6,574 (2.5)	
Infertility	3,003 (14.9)	12,773 (4.8)	<0.001
Pelvic pain	8,703 (43.0)	67,851 (25.7)	<0.001
Laparoscopic surgeries	4,277 (21.2)	4,476 (1.7)	<0.001
Danazol	1,863 (9.2)	1,556 (0.6)	<0.001
Estrogen (cream)	354 (1.8)	1,050 (0.4)	<0.001
Estrogen (tablet)	7,198 (35.6)	54,505 (20.7)	<0.001
Progestin (injection)	5,799 (28.7)	51,428 (19.5)	<0.001
Progestin (tablet)	9,717 (48.1)	68,330 (25.9)	<0.001

SD, standard deviation.

Patients in the endometriosis group were more likely than the controls to be diagnosed with migraine during the study interval (985 endometriosis cases [4.9%] *vs.* 7,701 controls [2.9%], OR = 1.70, 95% CI [1.59, 1.82], p<0.001; Table 2). Among the 985 endometriosis patients suffering from co-morbid migraine, a majority experienced migraines after their endometriosis diagnosis (776 cases [78.8%]) rather than before their diagnosis (209 cases [21.2%]). For those women who experienced migraines after their endometriosis, the mean duration between the two was 1,477±953.6 days, compared to the mean duration of 490±410.5 days for migraine occurring before endometriosis. If we considered only those migraines occurring near the time period at which endometriosis was diagnosed (from 1 year before the endometriosis diagnosis date to 1 year after the end date), a similar temporal relationship would be observed (after vs. before: 68.5% vs. 31.5%). We used the same strategy with a two-year period (from 2 years before the endometriosis diagnosis date to 2 years after the end date) and the temporal relationship was not affected (after vs. before: 66.5% vs. 33.5%). As shown in Table 2, both multivariate regression model 1 (OR = 1.36, p<0.001) and model 2 (OR = 1.37, p<0.001) independently displayed co-morbid relationships between endometriosis and migraines.

**Table 2:** Odds ratio values of migraine, including independent variables.

Independent variable	Odds ratio	95% CI	P-value
Model 1: Adjusted for age, diagnoses of infertility and pelvic pain, and treatments of laparoscopic surgeries			
Endometriosis	1.36	1.26–1.46	<0.001
Infertility	1.16	1.07–1.27	0.001
Pelvic pain	1.88	1.80–1.96	<0.001
Laparoscopic surgeries	1.11	0.99–1.24	0.065
Model 2: Adjusted for age, and danazol, estrogen, and progestin medications			
Endometriosis	1.37	1.27–1.47	<0.001
Danazol	1.36	1.16–1.58	<0.001
Estrogen (cream)	1.61	1.30–1.98	<0.001
Estrogen (oral)	1.27	1.21–1.34	<0.001
Progestin (injection)	1.23	1.17–1.30	<0.001
Progestin (oral)	1.31	1.24–1.37	<0.001

CI, confidence interval.

## Discussion

The results of this study indicated that migraines are 1.70 times more common in women with endometriosis than in those without the disease. This finding supports previous observations that endometriosis and migraines share a number of symptoms and risk factors. In addition, migraines were more frequently reported in women experiencing pelvic pain, a common symptom of endometriosis, than in women without pelvic pain (OR = 1.88, adjusting for endometriosis diagnoses). In a previous report, Karp et al. concluded that pelvic pain was an independent predictor of migraine [22]. Indeed, our data compliment this conclusion and demonstrate that endometriosis is also an independent predictor of migraine (OR = 1.36; adjusting diagnoses of pelvic pain).

A majority of the women experienced migraines after their endometriosis diagnosis rather than before their diagnosis (78.8% vs. 21.2%), even though we only considered migraines occurring near the time period at which endometriosis was diagnosed, or from 1 or 2 years before the endometriosis diagnosis date to 1 or 2 years after the end date. However, because of the longer mean duration between endometriosis diagnoses and follow-up onsets of migraines (after vs. before: 1,477 days vs. 490 days), the definition of the time period would filter out more cases of migraines occurring after the endometriosis diagnoses.

A number of aspects of the pathophysiological pathways believed to underlie endometriosis may explain its apparent relationship with migraine. For example, it has been proposed that the activation of sensory fibers within ectopic endometrial tissue (similar to an endometriosis lesion) can lead to neuronal hyperactivity throughout the central nervous system [24]. This phenomenon appears to be independent of nerve injury [25]. It is also possible that an excessive number of activated and degranulating mast cells within the endometriosis lesions or internal nerve structures can induce the release of a host of proinflammatory and algesic mediators. These mediators may then sensitize primary afferent meningeal nociceptive neurons [26], [27], which cause hypersensitivity and hyperalgesia, and potentially trigger the migraine attacks [7].

Our results also showed endometriosis patients had significantly greater hormone use than control subjects. Women with endometriosis were more likely to have been treated with injected or oral progestin than women without endometriosis. This finding was not surprising since progestin is a well-documented treatment in the management of endometriosis. In a similar vein, it was not surprising that the percentage of women using danazol was higher among those with endometriosis than among those without the disease. Although prescribed estrogen has been implicated in the pathogenesis of endometriosis, it is possible that the patients in our study may have been treated with this hormone for menorrhagia, dysmenorrhea or other health problems. In addition, transient menopause, a potential side effect of endometriosis therapy, is frequently treated with a hormone regimen consisting of estrogen.

Estrogen and other hormonal treatments have been shown to trigger migraines and migraine-related aura [28]. Consequently, migraine has been reported as an adverse side effect of estrogen use [29]. Oral estrogen can also worsen the headaches in postmenopausal migraine sufferers [30]. Endogenous variations in female hormone levels associated with the menstrual cycle and menstruation have also been shown to contribute to migraines [31], [32], [33]. Thus, it is reasonable to conclude that hormones prescribed for the treatment of endometriosis may influence the onset and severity of migraines. In the current study, a greater proportion of patients in the danazol medication group suffered from migraines than patients without this medication (OR = 1.36; after controlling for diagnoses of endometriosis). A similar association was observed under other hormone therapy conditions. However, differences in administration routes or types of female hormones may contribute to the conflicting results among studies. Changing the type of hormone or route of admission, such as switching to transdermal estrogen, is recommended if migraines occur as a side effect of hormone treatment [29]. The results of the current study did not support the protective effect of estrogen cream against migraine, but it would be difficult to reach a conclusion due to the small number of women in this hormonal group.

Our data demonstrated that endometriosis is still an independent predictor of migraine (OR = 1.37), after adjustment of hormone medications. Since patients with endometriosis had a higher probability of being treated with hormones, and both migraines and hormone therapy are associated with a higher incidence of cardiovascular disease [34], [35], we recommend that physicians screen endometriosis patients for migraines before prescribing hormone therapy. Although the use of female hormone therapies is associated with a higher risk for migraines, additional studies are needed to determine if this is a causal relationship.

The strengths of the current study include the use of a large population-based medical claims dataset, the inclusion of hormone treatments as a variable, and the independent regression models; however, we also acknowledge that this study had several limitations. First, the disease diagnoses were coded according to the ICD9-CM (for example, 346.0x, 346.1x, and 346.9x for migraine, and 617.x for endometriosis) and obtained from administrative claims reported by hospitals or clinics, which may be considered less accurate than clinical diagnoses by standard criteria. According to a prior survey [36], only 21.2% of endometriosis patients had surgical validation of the disease, and physicians in the outpatient clinic failed to appropriately diagnose approximately 23% of migraine sufferers. Furthermore a prevalence of 12% was shown in a subset of asymptomatic patients indicated for sterilization, and the overall prevalence among the women undergoing laparoscopy was 32.5% [37]. Nevertheless, a small proportion of subjects might have been under-diagnosed or misdiagnosed, and this constitutes a limitation of the current study. Despite these potential drawbacks, the data remain representative, as the Bureau of NHI routinely and randomly sampled a fixed percentage of claims from every contracted medical institution. Moreover, an independent group of physicians and gynecologists reviewed the charts and validated the diagnoses of endometriosis and migraines. Furthermore, the migraine diagnosis did not interfere with the endometriosis diagnosis and vice versa. Although headaches may be a side effect of hormone treatment used for endometriosis, as supported by our observation that 78.8% of migraines occurred following the diagnosis of endometriosis, our study still found that the prevalence of migraines was 37% higher in endometriosis patients (95% CI = 1.27∼1.47), even after adjusting for hormone therapies. Therefore, the potential for diagnostic deviation failed to influence the co-morbid relationship between endometriosis and migraines. A second limitation of the current study may be patient compliance. For example, medical chart evidence of the prescription of female hormones (or other drugs) does not verify that the drugs were actually taken by the patient. Therefore, our results may have underestimated the effects of medication on our subjects. Third, the administrative claims data from the NHIRD did not include detailed personal information (e.g., body mass index, living habits, or laboratory test results), which may add confounding factors to the relation of migraine or endometriosis. Fourth, the prevalence of migraines in this cohort was 3.1%, which is much lower than the 14.4% estimated prevalence for the community [13]. A previous study suggested that many patients with migraines fail to seek medical help [38], which may explain the discrepancy in our findings. Nonetheless, we should use caution when applying our conclusions to the community population as a whole. Fifth, we did not control all the potential risk factors of endometriosis and migraine which might influence their co-morbidities. A final limitation of our study pertains to the treatments used for women diagnosed with endometriosis. Transient menopause is a common consequence of the management of endometriosis and is triggered either by the surgical ablation of the ovarian function or the use of menopause-inducing medications, such as gonadotropin releasing hormone (GnRH) agonist. Unfortunately, we were unable to determine whether the patients in our study were being treated with GnRH, as the NHI Bureau did not cover this prescription.

In conclusion, the results of this population-based cohort study support the existence of a co-morbid relationship between migraine and endometriosis. This co-morbid association exists after adjusting the influences of pelvic pain symptoms or hormone therapies on migraine. Although endometriosis is associated with a higher risk of migraines, additional studies are needed to delineate the pathophysiological pathways underlying this co-morbid relationship.
